# Discrepancies in the Diagnosis of Congenital *Toxoplasma gondii* Infection Between B1 Gene Semi-Nested Polymerase Chain Reaction and Serological Analyses

**DOI:** 10.3390/microorganisms13030601

**Published:** 2025-03-05

**Authors:** Akiko Uchida, Kenji Tanimura, Keisuke Shirai, Mariko Ashina, Kazumichi Fujioka, Ichiro Morioka, Miwa Sasai, Masahiro Yamamoto, Hideto Yamada

**Affiliations:** 1 Department of Obstetrics and Gynecology, Kobe University Graduate School of Medicine, Kobe 650-0017, Japan; akikou@med.kobe-u.ac.jp (A.U.); taniken@med.kobe-u.ac.jp (K.T.); 2 Department of Pediatrics, Kobe University Graduate School of Medicine, Kobe 650-0017, Japan; ksk1024@med.kobe-u.ac.jp (K.S.); marikoa@med.kobe-u.ac.jp (M.A.); fujiokak@med.kobe-u.ac.jp (K.F.); 3 Department of Pediatrics and Child Health, Nihon University School of Medicine, Tokyo 173-8610, Japan; morioka.ichiro@nihon-u.ac.jp; 4 Department of Immunoparasitology, Research Institute for Microbial Diseases, Osaka University, Suita 565-0871, Japan; m-sasai@biken.osaka-u.ac.jp (M.S.); myamamoto@biken.osaka-u.ac.jp (M.Y.); 5 Laboratory of Immunoparasitology, WPI Immunology Frontier Research Center, Osaka University, Suita 565-0871, Japan; 6 Department of Immunoparasitology, Center for Infectious Disease Education and Research, Osaka University, Suita 565-0871, Japan; 7 Center for Advances Modalities and Drug Delivery Systems, Osaka University, Suita 565-0871, Japan; 8 Center for Recurrent Pregnancy Loss, Teine Keijinkai Hospital, Sapporo 006-0811, Japan

**Keywords:** B1 gene, congenital infection, diagnosis, newborns, polymerase chain reaction, pregnancy, screening, serological test, spiramycin, *Toxoplasma gondii*

## Abstract

Congenital *Toxoplasma gondii* (*T. gondii*) infection, which can be caused by a primary *T. gondii* infection during pregnancy, results in severe neurological sequelae in affected children. We have been conducting a prospective cohort study since January 2019 on pregnant women who were suspected of having primary *T. gondii* infection based on serological tests. In this study, congenital infection was diagnosed using semi-nested polymerase chain reaction (PCR) to detect the B1 gene in the body fluids of newborns. Up until December 2023, forty-one newborns born to mothers suspected of having primary *T. gondii* infection during pregnancy underwent B1 gene semi-nested PCR tests and anti-*T. gondii* immunoglobulin (Ig) G and IgM measurements of their blood samples. Eight newborns showed no clinical symptoms of congenital *T. gondii* infection; however, they were diagnosed with congenital *T. gondii* infection according to positive PCR results. However, none of the eight infants eventually exhibited any sign of congenital infection, as their serum samples tested negative for anti-*T. gondii* IgM and IgG until 12 months of age. Therefore, clinicians should consider discrepancies in the diagnosis of congenital *T. gondii* infection between PCR tests using body fluids of newborns and serological tests during their infantile period.

## 1. Introduction

*Toxoplasma gondii* (*T. gondii*) is a globally widespread zoonotic parasite, with infections prevalent in both humans and animals. *T. gondii* infection can lead to severe illness in individuals of all ages, particularly in immunocompromised patients and neonates.

Primary infection during pregnancy causes congenital *T. gondii* infection. Rarely, reactivation or reinfection of *T. gondii* may result in congenital *T. gondii* infection in immunocompromised pregnant women [1]. Congenital *T. gondii* infection results in chorioretinitis, intracranial calcification, hydrocephalus, and mental retardation in affected children. The incidence rate of congenital *T. gondii* infection varies by country and screening methods. For example, it is 3.4:10,000 births in France, 5—23:10,000 births in Brazil, 1:10,000 births in the USA [2], and 0.13—1.1:100,000 births in Japan [3].

On the other hand, accurate detection of primary *T. gondii* infection in pregnant women and prophylactic measures against fetal infection, including acetyl-spiramycin or spiramycin (SPM) treatment and fetal or neonatal therapy with pyrimethamine and sulfadiazine (P/S), reduce the incidence and severity of neurological sequelae of congenital toxoplasmosis [4,5]. In addition, neonatal therapy with P/S may have mild to moderate adverse effects, including bone marrow suppression, and tolerance to the therapy is generally reported to be good [6]. Therefore, neonatal therapy with P/S is recommended not only for symptomatic newborns but also for asymptomatic ones [7]. There is no doubt that effective maternal screening for detecting newborns at high risk of congenital *T. gondii* infection and comprehensive examinations for diagnosing congenital *T. gondii* infection in newborns are necessary. A nationwide survey on maternal screening for mother-to-child infections in Japan conducted in 2011 revealed that serological screening for *T. gondii* infection was performed in 48.5% of facilities [8]. Furthermore, SPM therapy for prophylaxis of transplacental transmission of *T. gondii* was covered by insurance in Japan in 2018. Therefore, the performance rate of maternal serological screening for congenital *T. gondii* infection may have increased. In addition, only one case of congenital *T. gondii* infection was reported in 2011 when 1,050,807 babies were born in Japan [3]. It is speculated that there may be overlooked cases of congenital *T. gondii* infection in Japan.

We have been conducting a prospective cohort study since April 2005 to assess the efficacy of maternal screening using anti-*T. gondii* immunoglobulin (Ig) G avidity measurement and a multiplex nested PCR assay that targets B1, cyclin-dependent kinase, SAG5E, and bradyzoite surface antigen 4 genes [4,5,9,10]. In our previous reports, we have demonstrated that our maternal screening for detecting pregnant women at high risk of congenital *T. gondii* infection, followed by prophylactic maternal therapy with SPM and P/S therapy for neonates with confirmed congenital infection, was effective in reducing the incidence and severity of long-term neurological sequelae in affected children [4,5].

Not only effective maternal screening but also accurate diagnostic methods for congenital *T. gondii* infection in neonates are important because they allow affected neonates to receive P/S neonatal therapy, which can improve their outcomes [4,5,10]. Methods for diagnosing congenital *T. gondii* infection in neonates include serological tests, such as anti-*T. gondii* IgM and IgA measurements from cord blood or neonatal peripheral blood [11,12], and PCR tests detecting *T. gondii* DNA in body fluids [13,14]. However, regarding *T. gondii* DNA PCR tests, there is no established method and no commercial kit; each laboratory uses its own method. Therefore, there is significant heterogeneity between laboratories, making it difficult to evaluate the diagnostic accuracy of PCR tests [15,16,17].

Further, we have been conducting another prospective cohort study since January 2019 to investigate the efficacy of the B1 gene semi-nested PCR assay for diagnosing congenital *T. gondii* infection.

According to the American Academy of Pediatrics (AAP) guidelines [13] and the manual of pregnancy management for *T. gondii* infection released by a Japanese study group [14], congenital *T. gondii* infection is diagnosed based on persistent serum anti-*T. gondii* IgG positivity in an infant after 12 months of age. Newborns are diagnosed with congenital *T. gondii* infection when they test positive for serum anti-*T. gondii* IgM, and *T. gondii* DNA is detected using PCR in their body fluids, including blood, urine, and spinal fluid, or when both clinical manifestations of congenital toxoplasmosis and serum anti-*T. gondii* IgG positivity are observed in neonates born to mothers with primary *T. gondii* infection during pregnancy.

Herein, we report eight newborns in our prospective cohort study who exhibited positive results for B1 semi-nested PCR in their blood but negative serum anti-*T. gondii* IgM and IgG during their infantile period.

## 2. Materials and Methods

### 2.1. Study Design and Participants

This prospective cohort study enrolled pregnant women who were referred to Kobe University Hospital due to suspected primary *T. gondii* infection from January 2019 to December 2023. This study followed the principles of the Declaration of Helsinki and was approved by the Institutional Review Board (IRB) of Kobe University Hospital (Reference no. B200362), and all participants provided written informed consent.

### 2.2. Procedures

**Figure 1:**
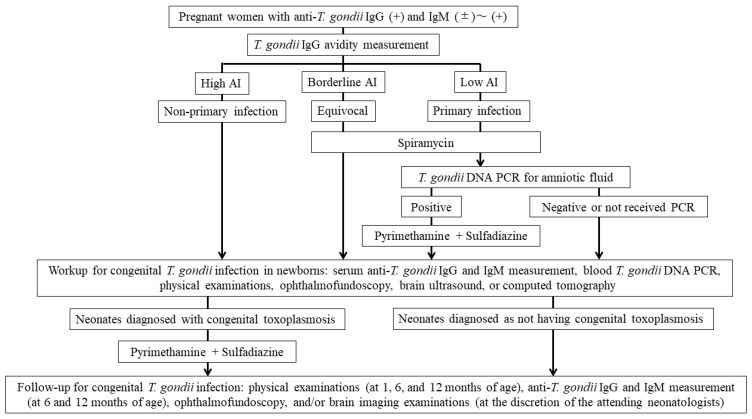
The maternal screening, neonatal evaluation, and follow-up algorithm for congenital toxoplasmosis in this study. Abbreviation: *T. gondii*, *Toxoplasma gondii*; Ig, immunoglobulin; AI, avidity index; PCR, polymerase chain reaction.

The maternal screening and neonatal evaluation and follow-up algorithm for congenital toxoplasmosis in this study is shown in Figure 1.

Pregnant women were referred to the Kobe University Hospital from January 2019 to December 2023 due to positive (>10.5 IU/mL until March 2020 and >3.0 IU/mL since April 2020) anti-*T. gondii* IgG levels (Access Toxo IgG, Beckman Coulter, Brea, CA, USA, until March 2020 and Toxo IgG Abbott, Abbott Laboratories, North Chicago, IL, USA, since April 2020) and positive (>1.0 IU/mL until March 2020 and >0.6 IU/mL since April 2020) or equivocal (0.8–0.99 IU/mL until March 2020 and 0.5–0.6 IU/mL since April 2020) IgM results (Access Toxo IgM, Beckman Coulter, Brea, CA, USA, until March 2020 and Toxo IgM Abbott, Abbott Laboratories, North Chicago, IL, USA, since April 2020). These pregnant women underwent serum anti-*T. gondii* IgG avidity index (AI) measurements (low: <30%, borderline: 30–35%, and high: >35%) (Daiichi Kishimoto Clinical Laboratories, Sapporo, Japan) and B1 semi-nested PCR analysis of their blood, which was performed as described in Section 2.4 (Research Institute for Microbial Diseases, Suita, Japan) [10]. Acute infection during pregnancy or periconceptional period was strongly suspected when the IgG AI was low, and thus women received SPM (9 million international units [IU]/day) until delivery [18]. Pregnant women with a borderline IgG AI were recommended to receive the SPM therapy. Chronic infection was suspected when IgG AI was high, and women did not receive SPM therapy unless they requested medication.

All newborns born to mothers who were enrolled in this study and delivered at Kobe University Hospital underwent serum anti-*T. gondii* IgG and IgM measurements, physical examinations, ophthalmofundoscopy, and brain ultrasound, or computed tomography. Additionally, newborns also underwent blood B1 semi-nested PCR tests when informed consent was obtained from their parents. According to our standard follow-up schedule, all newborns underwent physical examination at 1, 6, and 12 months of age, and serological tests for anti-*T. gondii* IgG and IgM at 6 and 12 months of age. Depending on the infants’ conditions or test results, additional physical and blood examinations, ophthalmofundoscopy, or brain imaging examinations at other ages were allowed at the discretion of the attending neonatologists. In addition, based on chronological age, psychomotor development was assessed as a developmental quotient using the Kyoto scale of psycho-logical development or Tsumori–Inage developmental test.

In this study, the diagnosis of congenital *T. gondii* infection was made by (1) positive results for serum anti-*T. gondii* IgM, (2) positive results for *T. gondii*-DNA PCR tests of neonatal body fluids, (3) the presence of both clinical manifestations of congenital toxoplasmosis and serum anti-*T. gondii* IgG positivity, and (4) the persistence of positive anti- *T. gondii* IgG after 12 months of age. In addition, the indications for neonatal P/S therapy in this study were (1) symptomatic congenital *T. gondii* infection, (2) positive results for anti-*T. gondii* IgM, and (3) positive results for anti-*T. gondii* IgG beyond 12 months of age. However, in this study, the positive results for blood B1 semi-nested PCR tests alone were not included as an indication for neonatal P/S therapy because the accuracy of the test was not determined.

### 2.3. Measurements of Serum T. gondii IgG Avidity

*T. gondii* IgG AI was measured using a commercially available kit (PLATERIA™ TOXO IgG AVIDITY, Bio-Rad, Hercules, CA, USA) according to the manufacturer’s instructions or in the commercial laboratory (The Daiichi Kishimoto Clinical Laboratories, Sapporo, Japan). Briefly, this method relies on the measurement of the avidity of *T. gondii* IgG. The use of an agent, including urea, dissociating the link between antigen and antibody in parallel with the usual technique of IgG measurement allows a comparison between the optical density (OD) obtained after dissociating agent action and that obtained without dissociating agent action (avidity index, % = urea-treated OD/untreated OD ×100). The AI is “low” when the antigen–antibody link is readily dissociated. An AI of <35% is considered “low” [5]; however, this value has not been standardized [5,14].

### 2.4. Detection of T. gondii Genomic DNA by B1 Semi-Nested PCR Tests

Blood samples from patients were centrifuged at 3000 rpm at 4 °C for 30 min, and the sera were discarded. Lysis buffer (300–1000 μL, 100 mM Tris-HCl; pH 8.0, 200 mM NaCl, 5 mM EDTA, 0.4% SDS) with protease K (50 μg/mL) was added to the clot. The samples were then incubated overnight at 55 °C. The samples were centrifuged at 10,000 rpm at 4 °C for 20 min, and the supernatants were collected for isolation of DNA using phenol–chloroform. DNA samples (100–200 ng) were used as PCR templates. Semi-nested PCR for the *T. gondii* B1 gene locus was performed using rTaq DNA polymerase (Toyobo, Osaka, Japan) with the following cycling protocol: 94 °C for 3 min, followed by 45 cycles at 94 °C for 30 s, 61 °C for 30 s, 72 °C for 1 min, and 72 °C for 2 min. The primer sequences used for semi-nested PCR were as follows: first PCR forward 5′- GGGGAAGAATAGTTGTCGCA-3′; second PCR forward 5′-GCTCTAGCGTGTTCGTCTCC-3′; and reverse 5′-GATCCTTTTG-CACGGTTGTT-3′. The product lengths of the first and second PCR sequences were approximately 450 bp and 200 bp, respectively [10].

## 3. Results

**Figure 2:**
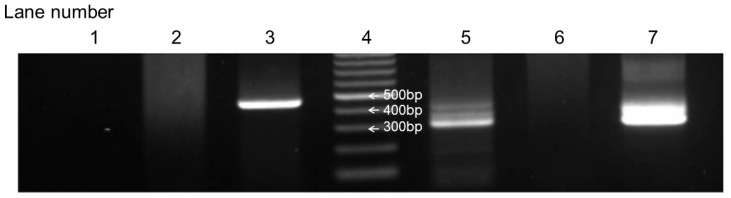
Positive B1 semi-nested PCR results of case 5 in Table 1. Lane 1: neonatal blood sample used for the first PCR analysis. Lane 2: negative control used for the first PCR analysis. Lane 3: positive control used for the first PCR analysis. Lane 4: marker. Lane 5: neonatal blood sample used for the second PCR analysis. Lane 6: negative control used for the second PCR analysis. Lane 7: positive control used for the second PCR analysis.

From January 2019 to December 2023, this study enrolled 81 neonates born at Kobe University Hospital to mothers enrolled in this study. Of these, 41 neonates underwent B1 semi-nested PCR analysis of their blood, with informed consent obtained from their parents. The PCR results were positive in eight newborns. Figure 2 illustrates a positive result of the B1 semi-nested PCR analysis in case 5 in Table 1 as an example.

Table 1 presents eight mothers and their newborns who tested positive for the B1 semi-nested PCR analysis of the newborns’ blood. All eight pregnant women demonstrated positive anti-*T. gondii* IgM results. Of the eight pregnant women, two (cases 2 and 3) exhibited low anti-*T. gondii* IgG AI, two (cases 6 and 7) demonstrated borderline levels, and four (cases 1, 4, 5, and 8) showed high IgG AI.

The mothers of cases 2, 3, 6, and 7 received oral administration of SPM (9 million IU/day) until delivery. However, three pregnant women (cases 1, 4, and 5) discontinued SPM therapy, and one (case 8) did not receive SPM therapy due to the suspicion of chronic *T. gondii* infection based on high IgG AI results.

All six newborns, except for case 4 (32 GWs) and 5 (36 GWs), were born at term (38–40 GWs). All seven, except for case 6 (−1.74 S.D.), were appropriate for gestational age.

All eight newborns underwent ophthalmoscopy, cerebral ultrasound, and head computed tomography. However, these examinations revealed no abnormal findings associated with congenital toxoplasmosis. Moreover, all eight newborns tested positive for serum anti-*T. gondii* IgG but negative for anti-*T. gondii* IgM after birth and did not receive P/S therapy. All six newborns at 12 months of age tested negative for serum anti-*T. gondii* IgG except for two newborns (cases 4 and 5) whose mothers were non-Japanese and returned to their home countries.

## 4. Discussion

Our present report describes eight newborns who tested positive for *T. gondii* B1 gene semi-nested PCR analysis on their blood samples. These newborns could be diagnosed with congenital *T. gondii* infection based on the AAP guidelines [13]. However, none of the newborns exhibited clinical symptoms of congenital *T. gondii* infection, and all of them tested negative for serum anti-*T. gondii* IgM. Furthermore, the serum anti-*T. gondii* IgG test results were negative 12 months after birth in six infants (cases 1, 2, 3, 6, 7, and 8) who were followed up after 1 year of age.

Early therapeutic intervention with a combination of P/S is recommended for newborns with congenital *T. gondii* infection despite not exhibiting any clinical symptoms of congenital toxoplasmosis. This is because such intervention reduces long-term sequelae in affected children [19].

Therefore, comprehensive examinations for diagnosing congenital *T. gondii* infection in newborns are necessary. Anti-*T. gondii* IgM and IgA measurements of cord blood or neonatal peripheral blood are widely used for diagnosing congenital *T. gondii* infection in neonates because maternal anti-*T. gondii* IgM or IgA cannot cross the placenta. The sensitivity and specificity of anti-*T. gondii* IgM tests for diagnosing congenital *T. gondii* infection during the neonatal period are reported to be 44–81% and 88.8–100%, respectively. These of anti-*T. gondii* IgA tests are 52–92.7% and 64–100%, respectively [20]. Previous studies have demonstrated that anti-*T. gondii* IgA measurements for pregnant women and neonates are useful for maternal screening [21,22] and diagnosis of congenital *T. gondii* infection [11,12], respectively. However, in our present study, anti-*T. gondii* IgA antibody measurements were not performed because a commercial assay of the antibody is not available in Japan. On the other hand, a comparative immunoblot test for anti-*T. gondii* IgG and IgM analysis from mother–neonate pairs at birth can be used for diagnosing congenital *T. gondii* infection [23].

Additionally, when serological tests are negative, *T. gondii* detection by PCR tests is recommended due to their high sensitivity [24]. The B1 gene is the most commonly employed target in PCR tests for *T. gondii* because it is thought to be the best target gene for these tests [25]. However, a previous meta-analysis demonstrated heterogeneity in the sensitivity values of *T. gondii* PCR tests [26]. The variability may be attributed to differences in PCR methodologies, types of specimens, timing of sample collection, and maternal treatment against *T. gondii* infection. A previous study reported that the sensitivity of PCR targeting the B1 gene in the amniotic fluid was higher than that in the neonatal blood (57.9% vs. 18.8%) [27]. Another systematic review demonstrated that PCR tests, including different targets—mainly the B1 gene—for amniotic fluid yielded 85.1% sensitivity and 99.7% specificity for diagnosing congenital *T. gondii* infection [26].

As mentioned, early commencement of neonatal P/S therapy is crucial to improve the outcomes of children with congenital toxoplasmosis. In addition, neonatal therapy with P/S may have mild-to-moderate adverse effects, primarily including bone marrow suppression, and tolerance to the therapy is generally reported to be good [6]. Therefore, overtreatment of neonates with suspected congenital *T. gondii* infection based solely on PCR-positive results may be acceptable. However, the prevalence of congenital *T. gondii* infection in Japan is thought to be extremely low [3], and the diagnostic accuracy of our B1 semi-nested PCR tests had not yet been determined. Therefore, it was feared that false-positive results of our PCR tests were more likely based on Bayes’ paradox [28], and the IRB of our institution did not allow treating newborns based on the positive results of our PCR tests alone. However, this methodology is not acceptable in countries where congenital *T. gondii* infection is not rare or where the diagnostic accuracy of PCR tests has been validated. In such countries and situations, newborns who test positive for PCR tests in their body fluids should receive neonatal P/S therapy. Furthermore, the persistence of anti-*T. gondii* IgG beyond 12 months of age in the absence of treatment is considered confirmatory for a diagnosis of congenital *T. gondii* infection [20]. In our present study, anti-*T. gondii* IgG was found to be negative by 12 months of age in six neonates who were positive for B1 semi-nested PCR. We believe that unnecessary P/S administration to newborns with overdiagnosed congenital *T. gondii* infection should be avoided whenever possible. Clinicians should pay attention to the interpretation of positive results from single PCR tests, which are not yet standardized.

A previous report from Europe revealed that over 70% (11 out of 15) of medical facilities used *T. gondii* PCR analysis that targets the B1 gene. False-positive results of PCR tests that target the B1 gene were observed in 4 (36.4%) of the 11 facilities [29].

Conversely, our previous prospective cohort study indicated that four of seven newborns born to pregnant women with a low AI were diagnosed with congenital *T. gondii* infection because they tested positive for multiplex nested PCR assays of *T. gondii* DNA in their blood, as well as in amniotic fluid at birth in two of the four newborns. However, all four newborns tested negative for serum anti-*T. gondii* IgG at 12 months of age [5]. The diagnosis of congenital *T. gondii* infection exhibited discrepancies between multiplex nested PCR tests for body fluids in newborns and serological tests during the infantile period.

We reported in our previous prospective cohort study the occurrence of congenital *T. gondii* infection only in mothers with low (<30%) anti-*T. gondii* IgG AI [5]. In our present study, newborns born to pregnant women with low (cases 2 and 3) or borderline (cases 6 and 7) anti-*T. gondii* IgG AI and those with high AI (cases 1, 4, 5, and 8) were suspected of having congenital *T. gondii* infection according to the positive B1 semi-nested PCR analysis results. Pregnant women who are suspected of having primary *T. gondii* infection generally receive prophylactic measures against the transplacental infection of *T. gondii* with maternal SPM therapy. However, maternal SPM therapy may prevent the occurrence of congenital *T. gondii* infection in pregnant women with a low AI. Thus, low maternal AI should be considered an important risk factor for congenital *T. gondii* infection.

On the other hand, it is reported that the risk of congenital *T. gondii* infection increases with gestational age. The maternal–fetal *T. gondii* transmission rate in maternal primary *T. gondii* infection is 2.2% at 6 GWs, but the rate increases to 23.0% at 18 GWs and 56.0% at 30 GWs [30]. In contrast, the severity of congenital toxoplasmosis is inversely proportional to gestational age [31]. In our study, all seven cases with positive results for the B1 semi-nested PCR test, except for case 3 whose anti-*T. gondii* IgM positivity was identified at 18 GWs, were suspected of having primary maternal infection during the first trimester or preconception period. Therefore, in this situation, it may be unlikely that newborns who did not have clinical symptoms of congenital toxoplasmosis at birth had the infection.

Conversely, in our previous prospective cohort study, one neonate was diagnosed with congenital toxoplasmosis because his mother was suspected of having a primary *T. gondii* infection during pregnancy (AI of 23% at 28 gestational weeks) and because he had intracranial calcifications and anti-*T. gondii* IgG positivity at 18 months of age [5]. However, the neonate tested negative for both serum anti-*T. gondii* IgM and multiplex nested PCR for *T. gondii* DNA in his blood at birth. These findings indicate that serum anti-*T. gondii* IgM and PCR tests for *T. gondii* DNA at birth may yield false-negative results.

This study had several limitations. First, the total number of participants was relatively small, and about half (40/81) of the newborns did not receive B1 semi-nested PCR tests on their blood because their mothers delivered at other hospitals or their parents refused. Second, because no newborn had congenital *T. gondii* infection during the study period, the diagnostic accuracy of the PCR tests cannot be determined. Third, we used a single PCR method, i.e., B1 semi-nested PCR, so we could not evaluate the utility of this PCR method by comparison with other PCR methods.

However, this study may provide useful information for clinical practitioners in perinatal medicine.

## 5. Conclusions

At present, standardized PCR methods for confirming congenital *T. gondii* infection remain unavailable [29]. Therefore, clinicians should be aware that diagnosing congenital *T. gondii* infection using a single test, such as PCR for detecting *T. gondii* DNA in the body fluid of newborns, may result in an inaccurate diagnosis. We believe that combination therapy with P/S should be administered to children accurately diagnosed with congenital *T. gondii* infection through multiple types of tests. Further, developing standardized *T. gondii* PCR assays is warranted.

## Figures and Tables

**Table 1:** Eight newborns who tested positive for B1 nested PCR analysis of blood samples and their mothers.

No	Maternal Anti- *T. gondii* IgM, IU/mL (GWs)	Maternal Anti- *T. gondii* IgG AI (GWs)	Maternal Therapy (GWs)	Birth Weight (GWs)/Gender	Clinical Manifestations of Congenital Toxoplasmosis	Anti-*T. gondii* IgG/IgM in Newborns’ Serum After Birth (Days of Age)	Anti-*T. gondii* IgG/IgM in Children’s Serum (Months of Age)	Outcome
1	2.6 * (15)	48% ^※^ (17)	Spiramycin (17–20)	2664 g (38) /female	None	Positive (0)/ Negative (0)	Negative (8)/ Negative (8)	5 years old; normal development
2	2.5 * (12)	20% ^※^ (13)	Spiramycin (12–38)	3126 g (38) /male	None	Positive (35)/ Negative (35)	Negative (12)/ Negative (12)	5 years old; normal development
3	6.1 ^#^ (18)	21% ^※^ (23)	Spiramycin (23–38)	2788 g (38) /male	None	Positive (0)/ Negative (0)	Negative (6)/ Negative (6)	2 years old; normal development
4	0.83 ^#^ (13)	59% ^※^ (16)	Spiramycin (16–18)	1908 g (32) /female	None	Positive (0)/ Negative (0)	N.D.	1 year old; N.D.
5	2.2 ^#^ (9)	89% ^※^ (11)	Spiramycin (10–13)	2594 g (36) /male	None	Positive (0)/ Negative (0)	N.D.	1 year old; N.D.
6	2.6 ^#^ (8)	55.4% ^†^ (12)	Spiramycin (12–40)	2578 g (40) /male	None	Positive (0)/ Negative (0)	Negative (7)/ Negative (7)	1 year old; normal development
7	2.76 ^#^ (11)	33% ^※^ (12)	Spiramycin (12–39)	3402 g (39) /female	None	Positive (0)/ Negative (0)	Negative (6)/ Negative (6)	1 year old; normal development
8	0.64 ^#^ (13)	62% ^※^ (17)	None	3144 g (40) /male	None	Positive (0)/ Negative (0)	Negative (11)/ Negative (11)	1 year old; normal development

Notes: * Anti-*T. gondii* IgM measured until March 2020 (positive: >1.0 IU/mL). ^#^ Anti-*T. gondii* IgM measured after April 2020 (positive: > 0.6 IU/mL). ^※^ Anti-*T. gondii* IgG AI measured by the Daiichi Kishimoto Clinical Laboratories (low: <30%, borderline: 30–35%, high: >35%). ^†^ Anti-*T. gondii* IgG AI measured by the ARCHITECT Toxo IgG Avidity assay (low: <50%, borderline: 50–59.9%, high: ≥60%). Abbreviations: No, number; *T. gondii*, *Toxoplasma gondii*; Ig, immunoglobulin; GWs, gestational weeks; AI, avidity index; PCR, polymerase chain reaction; N.D., not determined.

## Data Availability

The original contributions presented in the study are included in the article, further inquiries can be directed to the corresponding author.

## Abbreviations

The following abbreviations are used in this manuscript:

AI	avidity index
Ig	immunoglobulin
PCR	polymerase chain reaction
P/S	pyrimethamine and sulfadiazine
SPM	spiramycin
*T. gondii*	*Toxoplasma gondii*
